# Visual search advantages in tactical decision-making of male soccer players: evidence from 3V2 and 3V3 scenarios

**DOI:** 10.3389/fpsyg.2026.1931011

**Published:** 2026-09-17

**Authors:** Yang Zhao, Shunding Hu, Peng Shi, Xiaosu Feng

**Affiliations:** 1 Department of Physical Education, Xinxiang University, Xinxiang, China; 2 School of Physical Education, Liaoning Normal University, Dalian, China; 3 School of Physical Education, Shandong University of Technology, Zibo, China

**Keywords:** decision making, eye movement, offensive tactics, soccer, visual search

## Abstract

**Objective:**

This study aims to explore the visual search advantages of male soccer players with higher training experience in two offensive tactical decision-making scenarios (3V2 and 3V3) and clarify the differences in players’ visual search strategies under different levels of tactical complexity.

**Methods:**

A 2 (athletic level: higher training experience group vs. lower higher training experience group) × 2 (stimulus material: 3V2 scenario vs. 3V3 scenario) experimental design was adopted. The higher training experience group consisted of 16 players participating in the China University Football Association (CUFA) matches, while the lower training experience group was composed of 16 students majoring in soccer. Eye movements were recorded using a Dikablis Professional head-mounted eye-tracker manufactured by Ergoneers (Germany), with a mean calibration error = 0.25°. The stimulus materials were first-person perspective videos of 3V2 and 3V3 offensive scenarios, adapted from real match clips of the Chinese Football Association Super League (CSL). Based on SPSS 23.0 software, two-way analysis of variance (two-way ANOVA) was employed for statistical analysis.

**Results:**

In both the 3V2 and 3V3 scenarios, the higher training experience group showed significantly better performance than the lower training experience group in terms of decision reaction time and decision rationality (*p* < 0.05), with a more reasonable visual search strategy. Specifically, in the 3V2 offensive tactical decision-making scenario, the higher training experience group had significantly more fixations and longer fixation duration on the gap and goal areas compared to the lower training experience group (*p* < 0.05). In the 3V3 offensive tactical decision-making scenario, the higher training experience group exhibited significantly more fixations and longer fixation duration on the gap and far offensive player areas than the lower training experience group (*p* < 0.05). Additionally, in the 3V3 offensive tactical decision-making scenario, the higher training experience group spent significantly longer total fixation time on the goal, gap, near offensive players, and far offensive players (*p* < 0.05).

**Conclusion:**

The higher training experience group possesses faster and more rational tactical decision-making abilities as well as a concise and reasonable visual search strategy. This study provides guidance for athletes’ cognitive and decision-making training.

## 1 Introduction

Visual search refers to the process by which the body’s brain selectively analyzes and processes characteristic information of external objects and makes reasonable decisions (Formenti et al., 2022). Based on the extended human motor skill performance processing model (Schneider and Shiffrin, 1977; Wickens and Carswell, 2021), the information processing process of the organism can be divided into three stages: information input, information processing, and instruction output. The effectiveness of the information input depends largely on the quality of the visual information input; the speed of information processing depends largely on the complexity of the input information and the consistency of the input information with the information stored in the memory system; the instruction output is mainly reflected in the agility and rationality of the decision-making (Shi et al., 2023). In the field of sports, numerous studies (Voss et al., 2010; Yang et al., 2026) have confirmed that the cognitive advantages of professional athletes can be reflected through their visual search ability. Specifically, the results show that professional athletes exhibit shorter reaction times and higher prediction accuracy in different decision-making tasks (Shi et al., 2023; Yang et al., 2026). The core source of this advantage lies in the efficient cognitive processing patterns they develop through long-term training, which enables them to more accurately locate key information areas and more quickly activate tactical experience templates in memory, thereby reducing the ineffective consumption of cognitive resources (Habekost et al., 2024; Lex et al., 2015). Therefore, exploring the decision-making mechanisms of athletes with extensive training experience is essentially an analysis of the synergistic patterns between their cognitive systems and visual search (Araújo et al., 2019), which is of great significance for optimizing the efficiency of sports decision-making, designing targeted cognitive training programs, and improving the accuracy of training monitoring.

Athletes’ visual search strategies need to be comprehensively judged based on the number of fixations and fixation duration on different Areas of Interest (AOIs) (Wolfe, 2020), and these two indicators are precisely the visualized carriers of the cognitive processing process. The number of fixations reflects the extraction range and screening frequency of the cognitive system for stimulus information. The fewer the number of fixations while covering more comprehensive information, the higher the accuracy of cognitive screening (Wolfe, 2020). Fixation duration, on the other hand, reflects the depth and difficulty of information processing by the cognitive system. If an athlete can complete the processing of key information in a relatively short period of time, it indicates that there are highly matched experience templates in their memory, which enables the rapid integration of information (Wolfe, 2020). It is particularly important to note that the interpretation of fixation indicators must take into account differences in cognitive levels. Based on Cognitive Load Theory (Zeitlhofer et al., 2026), the long fixation durations of athletes with extensive training experience may reflect the deep processing of critical information (e.g., rapid association with tactical experience), whereas those of athletes with limited training experience may indicate insufficient cognitive resources and difficulties in information extraction (Shi et al., 2023). Such cognitive differences constitute the core logical foundation for this study to compare the higher training experience group and the lower training experience group.

Soccer is characterized by high-intensity physical confrontation and rapid offensive and defensive transitions (Wang, 2015). The dynamism and complexity of its match scenarios place extremely high demands on players’ multiple object tracking ability. Players need to simultaneously process multi-dimensional information such as teammates’ positions, opponents’ defenses, changes in ball possession, and gap creation—this highly aligns with the core requirement of simultaneously monitoring, extracting, and integrating multiple dynamic targets in the multiple objects tracking theory (Shi et al., 2023). Based on the task classification framework of the multiple object tracking theory, visual search in soccer scenarios can be divided into single-target tasks (e.g., penalty kicks, corner kicks) and multiple-target tasks (e.g., offensive and defensive tactics). Single-target tasks involve simple external stimuli, and the cognitive system only needs to focus on single key information (e.g., the leg movements of a penalty taker); mature eye movement processing models have been developed in relevant studies (Wu et al., 2012; Vítor de Assis et al., 2021). In contrast, multiple-target tasks require the cognitive system to allocate resources to multiple dynamic targets simultaneously; making their processing far more difficult than that of single-target tasks—and this also represents the current focus and difficulty of research.

Existing studies related to multiple object tracking have confirmed that soccer players’ visual search strategies are directly associated with the quality of their tactical decision-making. McGuckian et al. (2020) found that the gaze characteristics of players when observing teammates’ runs and opponents’ gaps are significantly correlated with their passing success rate, which essentially reflects the explicit manifestation of multiple objects tracking ability influencing cognitive decision-making. Natsuhara et al. (2020) pointed out that high-level players can more efficiently track key targets such as off-the-ball opponents and receiving teammates. The reason lies in that their cognitive systems can quickly screen effective information through experience templates, thereby reducing interference from irrelevant stimuli. Aksum et al. (2020, 2021), in their studies on 11-a-side matches, further discovered that the gaze focus (soccer ball, opponents, ball-controlling players) of elite midfielders is highly consistent with the cognitive demands of offensive and defensive phases, and the duration of their gaze on opponents is significantly longer. This is a typical example of dynamically adjusting the allocation of cognitive resources according to task demands in the multiple objects tracking theory. However, the complexity of 11-a-side match scenarios makes the division of AOI difficult. Existing studies fail to accurately capture detailed information such as the on-the-ball/ off-the-ball status of players and their positional characteristics (Aksum et al., 2020; Aksum et al., 2021), resulting in an insufficiently in-depth analysis of the correlation between multiple object tracking ability and tactical decision-making. Based on this, Shi et al. (2023) proposed a research approach starting from local offensive and defensive scenarios. Among these, “3V2” (3 attackers vs. 2 defenders) and “3V3” (3 attackers vs. 3 defenders), as classic local tactical scenarios (Wang, 2015), have a clear number of targets and controllable scenario complexity. They not only meet the research requirements of dynamic target monitoring in the multiple objects tracking theory but also allow for precise division of AOIs (e.g., attacking players, defending players, gap areas). This provides an ideal scenario for analyzing the collaborative mechanism between the cognitive system and visual search.

Existing research conclusions on local tactics are inconsistent, but the core divergence essentially lies in the matching relationship between cognitive level and scenario complexity. Williams et al. (1994) found that experienced players can attend to more targets and maintain longer gaze durations, which reflect that their cognitive systems can improve multiple objects tracking efficiency through in-depth processing. However, Williams and Davids (1998) did not find experience-based differences in the 3V3 scenario. This may be because the cognitive load of this scenario exceeds the processing capacity of low-level players, while failing to pose sufficient challenges to high-level players—ultimately leading to convergent visual search characteristics between the two groups (Silva et al., 2022). Integrating the multiple object tracking theory and the cognitive load theory, this study proposes the following hypotheses:

### 1.1 Research hypothesis 1

Athletes in the higher training experience group adopt more economical and efficient visual search strategies, leading to faster and more rational tactical decision-making. This hypothesis is primarily grounded in the logic of experiential advantage: athletes in the higher training experience group develop richer tactical schema through long- term training, which reduces the waste of cognitive resources and enables them to process multi-target information economically and efficiently, thereby resulting in quicker and more rational tactical decisions.

### 1.2 Research hypothesis 2

There are inconsistencies in the visual search characteristics of “3V2” and “3V3” attack tactical decisions. As offensive difficulty increases, athletes’ cognitive load also rises, yet the higher training experience group can still maintain efficient visual search strategies. This hypothesis is mainly proposed based on task difficulty. The difference in scenario complexity between “3V2” and “3V3” leads to different processing requirements of the cognitive system. The “3V3” scenario requires simultaneous tracking of more defensive targets, resulting in a significantly higher cognitive load than “3V2”; thus, there are differences in visual search characteristics between the two scenarios. However, athletes in the higher training experience group, possessing greater cognitive resource reserves and richer experiential schema, can still maintain efficient multi-target tracking ability in the high cognitive load “3V3” scenario.

Based on the above, this study aims to analyze the visual search advantages of highly experienced soccer players in offensive tactical decision-making scenarios of “3V2” and “3V3,” and further explore the differences in their visual search strategies across distinct tactical decision-making contexts. Through this study, it is expected to explain the visual search characteristics and information processing mechanism of multi-target tracking in offensive tactical decision-making situations, and then to provide psychological guidance for players’ specialized cognitive training, athletic selection and training monitoring.

## 2 Methods

### 2.1 Participants

This study is an exploratory research involving the interaction of two factors. Traditionally, exploratory research has relatively loose requirements for the strict calculation of sample size. However, to further ensure the reliability of the results of the higher training experience group and the lower training experience group, and avoid problems such as insufficient test power for interaction effects and misjudgment of inter-group differences caused by unbalanced inter-group sample sizes or insufficient sample sizes, this study still supplemented the sample size calculation using G·Power 3.1.9.7 software. Drawing on Silva et al. (2022), this study extracted their effect size (*ES* = 1.21), set the alpha error (*α* err) at 0.05, the power (Power 1-*β*) at 0.80, and the ratio of the two groups at 1:1. The calculation showed that a total of 24 participants were required. Thirty-two male participants were recruited using convenience sampling and divided into a higher training experience group and a lower training experience group based on their athletic level and training experience. The higher training experience group consists of 16 players who participate in the China University Football Association (CUFA). The average age of the participants is (18.75 ± 0.56) years old, with a training duration of 5 to 8 years, and their athletic level is above the national second-level athlete. The lower training experience group is composed of soccer major students from the Liaoning Normal University. The average age of the participants is (19.5 ± 1.21) years old, with a training period of 1 to 3 years, and they do not have any athletic ratings. All participants reported being physically healthy, with normal vision, and without any physical or mental illnesses. In addition, the participants gave their written informed consent following the Declaration of Helsinki. The study was approved by the Ethics Committee of Liaoning Normal University (approval no. LL2023022).

### 2.2 Study design

Two-factor mixed experiment design uses 2 athletic levels (higher training experience group and the lower training experience group) × 2 stimulus materials (“3V2” and “3V3”). The variables between groups are athletic levels, and the variables within groups are stimulation materials. The study divides stimulus videos into five areas of interest (AOI): goal, gap, defensive player, near offensive player and far offensive player (see Figure 1). The sequential images converted from the video are detailed in Supplementary material 1.

**Figure 1:**
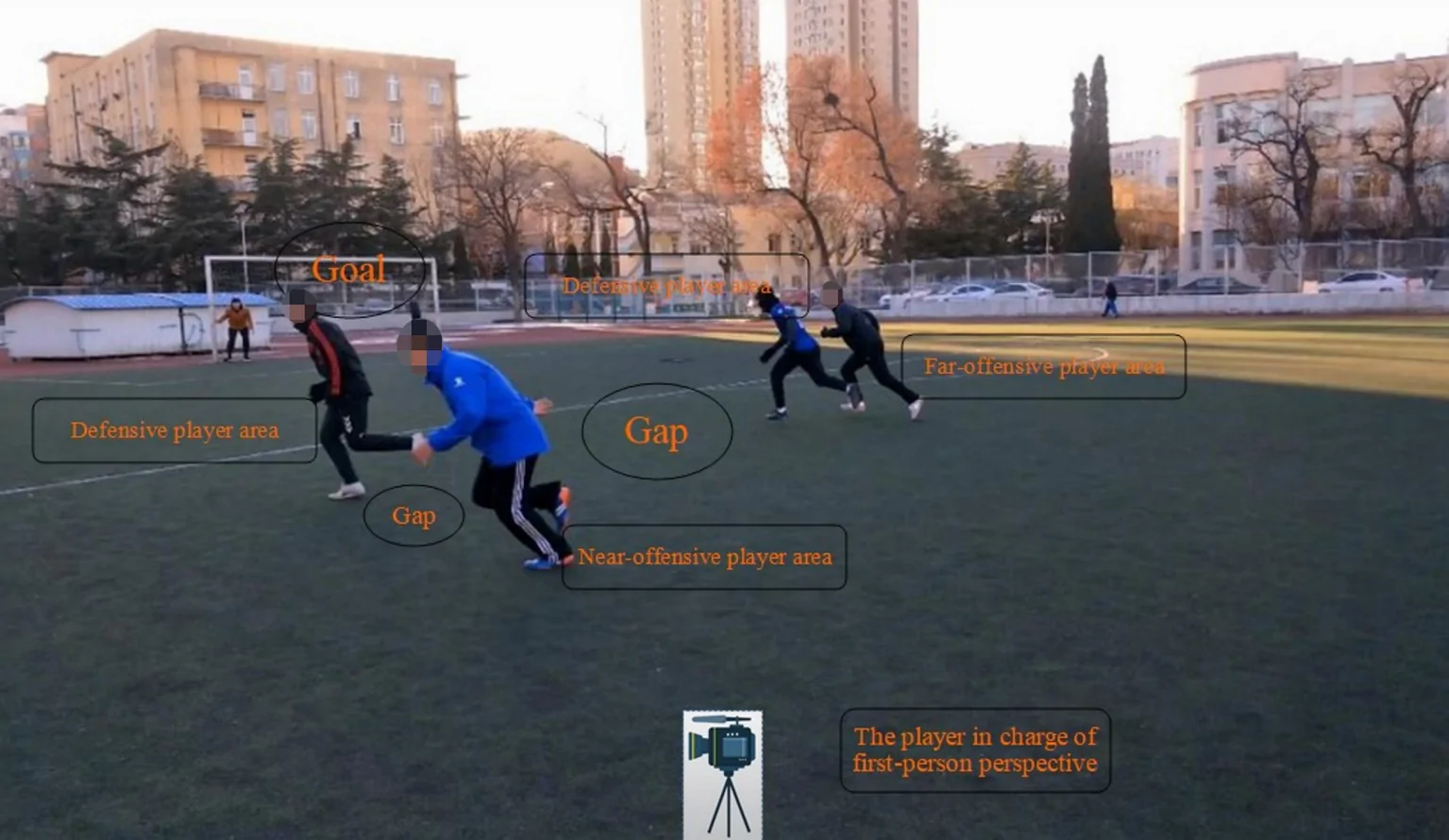
Schematic diagram of AOI.

In the stimulus videos of the study, the defined Areas of Interest (AOIs) for analyzing soccer players’ visual search characteristics are as follows: the Goal AOI refers to the soccer goal area in the videos, covering the goal frame, goal line, and the range within the goal area (penalty area), and it serves as the core target for the offensive team’s final attack; the Gap AOI denotes the unguarded spatial areas formed between the offensive and defensive teams in the videos, including static gaps (such as the spaces between defensive players in their formation) and dynamic gaps (such as the temporary spaces exposed when defensive players move), which act as key channels for the offensive team to break through the defense; the Defensive Player AOI refers to the areas covering the bodies of defensive players in the videos, including visual information like their positions and body movements, and it is a key reference for the offensive team to judge defensive pressure and defensive intentions; the Near offensive Player AOI refers to the offensive teammates who are close to the “first-person shooter” in the videos, usually those within a distance that allows direct passing or coordination, with their visual area including information such as their positions, running directions, and body signals (e.g., reaching out for the ball); and the Far offensive Player AOI refers to the offensive teammates who are far from the “first-person shooter” in the videos, including those on the opposite side of the defensive team who require long passes or large-scale transfers for coordination, and their visual area also covers information like positions and running trajectories, though with lower visual clarity compared to near offensive players.

This study, drawing on previous research (Gao et al., 2025; Shi et al., 2023), explores the mechanism of visual information processing from two dimensions: athletes’ tactical decision-making efficiency and visual search characteristics. Indicators reflecting tactical decision-making efficiency include tactical decision-making agility and tactical decision-making rationality. Tactical decision-making agility focuses on speed, referring to the ability to respond quickly in a dynamic environment. Tactical decision-making rationality focuses on quality, referring to whether a decision aligns with goals and objective conditions (Gao et al., 2025; Shi et al., 2023). In addition, indicators reflecting visual search characteristics include number of fixations and fixation duration.

### 2.3 Apparatus and stimulus materials

Dikablis Professional head-mounted eye tracker produced by Ergoneers in Germany was used to record the eye movement data of each participant. The sampling rate was 60 Hz and the tracking accuracy was 0.1° to 0.3°. In addition, two computers were selected for experimental operations. One of the computers equipped with D-Lab software is used to connect the eye tracker and record the participants’ observation process. The other computer, equipped with E-prime 3.0 software, is connected to a SHARP projector, which is used to play the “3V2” and “3V3” stimulus materials, as well as for the participants to respond with keystrokes and mark decision-making routes.

The stimulus materials selected in this study were excerpts from real “3V2” and “3V3” attack scenarios from the 2022 Chinese Super League (CSL). Thirty stimulus videos were captured using EV screen recording software, with 15 videos for each of the “3V2” and “3V3” attack scenarios. Additionally, one experienced soccer coach was invited to evaluate these videos, mainly retaining one video with similar attacking routes and player movements. Ultimately, 22 videos were obtained. This coach is a retired player from Shandong Taishan, a historically strong team of the CSL, and the head coach of the Liaoning Normal University CUFA team, with extensive professional and coaching experience. The screening criteria are as follows: (1) Tactical Typicality: Scenarios with clear tactical logic that conform to the core characteristics of “3V2 (3 attackers vs. 2 defenders)” and “3V3 (3 attackers vs. 3 defenders)” are prioritized for retention, while clips with ambiguous tactics (such as chaotic player movements and no clear offensive intent) are excluded; (2) Information Integrity: It is ensured that key elements in the videos (goal, gap, offensive players, defensive players) are clearly visible, without issues like camera obstruction or blurred images, so as to avoid influencing participants’ decisions due to information loss; (3) Movement Consistency: Videos with similar offensive routes are selected to reduce additional interference caused by excessive scenario differences, thereby ensuring the comparability of videos under the same condition. Finally, nine “3V2” videos and nine “3V3” videos were selected as the main materials for the test, while two “3V2” videos and two “3V3” videos were chosen as preparation materials.

Furthermore, to simulate real-match scenarios from a first-person perspective, this study invited 6 college students majoring in soccer to recreate the tactical scenarios from the aforementioned CSL match clips. The soccer skill level of these 6 student players was consistent with that of the novice group (soccer-major students) in this study, so as to minimize the interference of skill differences among performers on the authenticity of the scenarios. One player served as the ball-carrying offensive player and conducted first-person shooting with a Canon 6D camera equipped with a 50 mm fixed-focus lens. Clear requirements were specified before shooting: (1) The lens must fully capture the positions and movements of all offensive and defensive players, ensuring that the field of view is consistent with the actual observation field of the offensive players in the original CSL clips; (2) Camera stability should be controlled by means of holding the camera steadily with both hands and presetting the shooting distance, so as to reduce motion blur; (3) Camera parameters should be uniformly adjusted to ensure consistent brightness and contrast across all videos, avoiding the interference of light and shadow differences on participants’ visual judgment. The remaining 5 players, based on the movement trajectories, speeds, and interaction rhythms of players in the original CSL clips, played the roles of offensive and defensive players, respectively. Through rehearsals (2–3 rehearsals for each scenario), they ensured that the timing and routes of their movements were highly consistent with the tactical dynamic characteristics of the original clips.

Eventually, 18 formal test videos and 4 practice experiment videos were shot. Among them, there were 9 “3V2” offensive tactical videos (3 for each of the left, middle, and right routes) and 9 “3V3” offensive tactical videos (3 for each of the left, middle, and right routes). By evenly distributing the number of videos across different offensive routes, the representativeness of the spatial distribution of scenarios was ensured. All stimulus videos contained core tactical elements such as the goal, offensive players (teammates), and defensive players (opponents), with a duration controlled between 3 and 6 s. The time nodes were strictly aligned with the original CSL clips—starting when the opposing defensive players or the team’s own offensive players began to move, and ending when all players had completed their movements and the tactical actions temporarily came to a stop. This was done to ensure that the temporal dynamic characteristics of the videos were consistent with real- match scenarios.

### 2.4 Dependent variables

#### 2.4.1 Tactical decision-making efficiency

1. Tactical decision-making agility. The agility of participants’ tactical decision-making is reflected by the response time recorded via keystroke. The response time is measured from the start of the stimulus material playback to the time when the participant presses the key, and the unit is milliseconds.
2. Tactical decision-making rationality. In realistic soccer scenarios, there is a diversity of offensive tactical decision-making, involving various instant decisions such as passing, controlling, and shooting. However, it is often challenging to determine whether a particular decision is correct or incorrect. Therefore, this study refers to the research conducted by Shi et al. (2023) and introduces the rationality of tactical decision-making to assess the quality of participants’ decisions. Two sports psychology professors and two senior CUFA coaches were invited to form a scoring team. Prior to scoring, all experts received unified training to clarify the scoring criteria and operational specifications. A 5-point Likert scale was adopted for scoring, with the core evaluation basis being the comprehensive threat level of participants’ decisions to the goal. Specifically, it included three dimensions: tactical adaptability of decision-making choices (e.g., gap utilization, teammate position matching), offensive efficiency (e.g., whether to shorten offensive time, reduce passing times), and defensive breakthrough probability (e.g., whether to avoid core defensive areas, utilize defensive weaknesses). A higher threat level corresponds to a higher score. To avoid scoring bias, a blinded scoring design was implemented in this study. All stimulus videos were stripped of participants’ group identifiers, personal information, and relevant characteristic clues, presenting only the tactical scenarios themselves. Scoring experts completed the evaluation independently without knowing the participants’ group affiliations, and no communication regarding scores was conducted among the experts. The results of the expert scoring consistency test showed that the scores exhibited moderate consistency (Kendall’s coefficient of concordance *W* = 0.491, *χ*^2^ = 53.047, *p* < 0.01; Intraclass Correlation Coefficient ICC = 0.892, 95% Confidence Interval [0.785, 0.951], *p* < 0.01). Finally, the average score of the four experts was taken as the tactical decision-making rationality score for each participant when watching each video.

#### 2.4.2 Visual search data

A fixation was defined as a condition in which the eye remained stationary within a tolerance of movements up to 3° for a period greater than 100 ms (Panchuk and Vickers, 2006). In this study, the stimulus videos were divided into five AOIs: goal, gap, defensive players, near offensive players, and far offensive players. Fixations were assigned to corresponding AOIs frame-by-frame via manual labeling by one researcher, with a second researcher conducting cross-verification. Any discrepancies in labeling were resolved through group discussion to reach a consensus. The number of fixations and fixation duration in each AOI were collected using D-lab software.

Fixations serve as the fundamental primary judgment unit, which determines whether a period of gaze retention constitutes a valid fixation and acts as the underlying data source for all eye-movement statistical analyses. AOIs function as spatial classification tools used to categorize pre-screened fixations by spatial region. Firstly, all valid fixations are filtered from the massive real-time sampling data captured by the eye tracker based on fixation criteria, and the precise spatial coordinates corresponding to each fixation are extracted. Secondly, the coordinate of each fixation is matched and compared against the boundaries of all AOIs on the stimulus footage to identify which tactical partition the fixation falls within. Finally, data are aggregated by AOI group to calculate number of fixations and total fixation durations for each region separately.

1. Number of fixations. The number of fixations refers to the quantity of gaze occurrences within the aforementioned AOIs during the period from when the participant begins watching the video to when they press a key to make a decision (Friedman and Komogortsev, 2024; Klostermann and Moeinirad, 2020).
2. Fixation duration. Fixation duration refers to the total amount of time spent gazing within the aforementioned AOIs, from the moment the participant begins watching the video to the time they press a key to make a decision. It is the sum of the duration of each individual fixation. The unit for this is milliseconds (Friedman and Komogortsev, 2024; Klostermann and Moeinirad, 2020).

The selection of the above five AOIs is closely aligned with the core research objectives and the inherent nature of the tactical scenarios. The exclusion of potential AOIs such as touchlines and goal lines is essentially to avoid interference from irrelevant information, ensure the research focus, and enhance the validity of the conclusions.

### 2.5 Procedure

The participants were individually brought into the sports psychology laboratory in order, and their basic information (age, gender, sport level, exercise duration) was recorded. After entering the testing area, participants sat on a chair facing the projector, maintaining a fixed distance of approximately 3 meters from the projection screen. Researchers vertically adjusted the projection position according to the participants’ sitting height to ensure the center of the screen image was aligned with the participants’ line of sight, thereby avoiding any impact on visual information reception due to viewing angle deviations. Subsequently, the researchers assisted the participants in wearing the eye tracker, and adjusted the tightness of the headband manually to ensure the device fit the head without causing a sense of pressure. Then, a four-point calibration method was adopted to complete the instrument calibration, with the calibration accuracy controlled within the range of 0.1° ~ 0.3° (which complies with the standard accuracy range of the device). Immediately after calibration, a validation check was conducted for each participant: five randomly distributed visual targets were presented, and the eye tracker’s capture deviation of the target fixation positions was recorded. If the deviation of a single target exceeded 0.5°, recalibration was performed. During the experiment, if a tracking interruption lasting more than 100 ms occurred, the experiment was paused and recalibration was carried out. Additionally, the participant was instructed to place their dominant hand’s index finger on the spacebar of the computer playing the stimulus video, which would be used to initiate the playback of the video and make decision-making presses.

In order to improve the familiarity of the participants with the experimental process and operational tasks, each participant conducted a pre-experiment. During the pre-experiment, the researcher promptly instructed and corrected the improper operation of the participants until each participant was able to complete the experimental tasks independently. After participants were familiarized with the experimental process and operational tasks, the formal experiment was started. A “+” attention symbol was displayed in the center of a blank screen. The participant pressed the spacebar on their own to start playing the stimulus video. After watching the video, they pressed the spacebar again and used the mouse to mark the decision route. Additionally, the participant verbally reported the specific content of their decision to the researcher. If the participant failed to press the key to complete the decision within the specified time, the screen would switch to a blank interface, and this decision would be deemed invalid with a score of 0. The decision reaction time for this invalid trial is recorded as the total playback duration of the stimulus video. In this study, the 18 test videos used were presented in a fully randomized order using the randomization function in E-Prime 3.0. Pseudo randomization was performed with a fixed random seed of 20,250,211 to avoid interference with experimental results caused by order effects. There were 18 test videos for each participant, and the test time was about 5 min.

### 2.6 Statistical analysis

In this study, D-lab software was used to record participants’ response times and gaze-related data, which were then imported and saved in Excel. Furthermore, the data in Excel was imported into SPSS 23.0 software, where the scores of tactical decision-making rationality were matched with the response times and gaze data according to each participant’s order of watching the videos. During the data preprocessing phase, missing values were handled using the linear interpolation method, with the interpolation approach set as linear and 13 trials (2.26% of the total) undergoing interpolation. In addition, the threshold for trial interruption was defined as a visual tracking loss lasting more than 100 ms. The eye movement data derived from interpolation were incorporated into the corresponding AOIs, and then combined with the raw collected data for the statistical analysis of fixation count and fixation duration indicators.

Descriptive statistics for continuous variables were conducted using mean (*M*) and standard deviation (*SD*). The Shapiro–Wilk test was used in combination with Q-Q plots and P–P plots to test the normality of the data, and the results showed that the relevant data exhibited an approximately normal distribution. For the variables of tactical decision-making and visual search, a two-way mixed analysis of variance (ANOVA) model with athletic level (higher training experience group vs. lower training experience group) × tactical scenario (3V2 vs. 3V3) was constructed, with participants treated as a random factor for repeated measures. The Levene’s test was used to verify the homogeneity of variances of the data; if the homogeneity of variances was violated, the Welch correction was applied to adjust the test statistics. Mauchly’s test of sphericity was employed to verify the sphericity assumption of the data. If the sphericity assumption was violated, a correction method was selected based on the epsilon (*ε*) value to ensure the accuracy of the statistical results: the Greenhouse–Geisser correction was adopted when *ε* < 0.75, and the Huynh-Feldt correction was used when *ε* ≥ 0.75. The EMMEANS statement in SPSS was used to estimate marginal means for conducting simple effect analysis, and the Bonferroni correction method was employed to adjust the error rate of multiple comparisons. In addition, this study uses *η*^2^ to represent the effect sizes of main effects and interaction effects, and supplements the effect sizes of simple effect tests with Cohen’s *d*. GraphPad Prism 8 software was utilized for the visual presentation of the results.

In addition, post-hoc observed power was employed in the present study to examine the robustness of the significant interaction effect (Castillo et al., 2026). The ANOVA effect size *f* was calculated from the observed partial *η*^2^ of the interaction effect, with *f* = $\sqrt{\frac{\eta 2}{1-\eta 2}}$ . Post-hoc observed-power analysis was performed using G*Power 3.1.9.7 with the following parameters: *α* = 0.05, total sample size *n* = 32, number of between-participants groups = 2, number of repeated-measures levels = 2, and correlation among repeated measures set to 0.5. Power ≥ 0.80 was regarded as an acceptable threshold, indicating that the sample size was sufficient to reliably detect the interaction effect and supporting robust results. Power values ranging from 0.7 to 0.8 were considered marginally acceptable; although slightly below the ideal threshold, the interaction effect remained statistically significant. Power < 0.7 indicated low statistical power, suggesting limitations in the sample size.

## 3 Results

### 3.1 Tactical decision-making efficiency

The main effect of athletic level is significant in terms of tactical decision-making agility (*F* = 20.572, *p* < 0.001, *η_p_^2^* = 0.391). The reaction time of tactical decisions made by the higher training experience group (2110.00 ± 433.67 ms) is significantly faster than that of the lower training experience group (2987.71 ± 739.80 ms). The main effect of stimulus materials is not significant (*F* = 3.377, *p* = 0.075, *η_p_2* = 0.095). The interaction between athletic levels and stimulus materials is not significant (*F* = 1.715, *p* = 0.200, *η_p_^2^* = 0.051). Further simple effect analysis showed that the tactical decision-making reaction time of the higher training experience group (2160.11 ± 193.52 ms) in the 3V2 scenario was significantly shorter than that of the lower training experience group (3292.22 ± 272.52 ms; *p* < 0.01, Cohen’s *d* = 4.790). Additionally, the tactical decision-making reaction time of the higher training experience group (2058.89 ± 202.86 ms) in the 3V3 scenario was significantly shorter than that of the lower training experience group (2683.21 ± 222.57 ms; *p* < 0.05, Cohen’s *d* = 2.932). Furthermore, there was no significant difference in the decision-making reaction time of the higher training experience group when watching 3V2 and 3V3 videos (*p* = 0.711). In contrast, the decision-making reaction time of the lower training experience group was significantly slower when watching 3V2 videos (3292.22 ± 272.52 ms) than when watching 3V3 videos (2683.21 ± 222.57 ms; *p* = 0.033, Cohen’s *d* = 2.234).

In terms of tactical decision-making rationality, the main effect of athletic level is significant (*F* = 31.668, *p* < 0.001, *η_p_^2^* = 0.497). The score of tactical decisions made by the higher training experience group (3.79 ± 0.29) is significantly higher than that of the lower training experience group (3.26 ± 0.33). The main effect of stimulus materials is not significant (*F* = 2.883, *p* = 0.099, *η_p_^2^* = 0.083). The interaction between athletic levels and stimulus materials is significant (*F* = 5.091, *p* = 0.031, *η_p_^2^* = 0.137). The post-hoc observed power for this interaction effect was calculated to be 0.742, falling within the marginally acceptable range, and the statistical results remain robust. Further simple effect analysis showed that the tactical decision-making score of the higher training experience group (3.61 ± 1.00) in the 3V2 scenario was significantly higher than that of the lower training experience group (3.28 ± 0.95; *p* < 0.05, Cohen’s *d* = 0.338); the tactical decision-making score of the higher training experience group (3.98 ± 1.02) in the 3V3 scenario was significantly higher than that of the lower training experience group (3.23 ± 0.91; *p* < 0.01, Cohen’s *d* = 0.776). In addition, the decision-making score of the higher training experience group (3.61 ± 1.00) when watching the 3V2 video was significantly lower than that when watching the 3V3 video (3.98 ± 1.02; *p* = 0.009, Cohen’s *d* = 0.366), while there was no significant difference in the decision-making score of the lower training experience group when watching the 3V2 and 3V3 videos (*p* = 0.696). Detailed results are shown in Figure 2.

**Figure 2:**
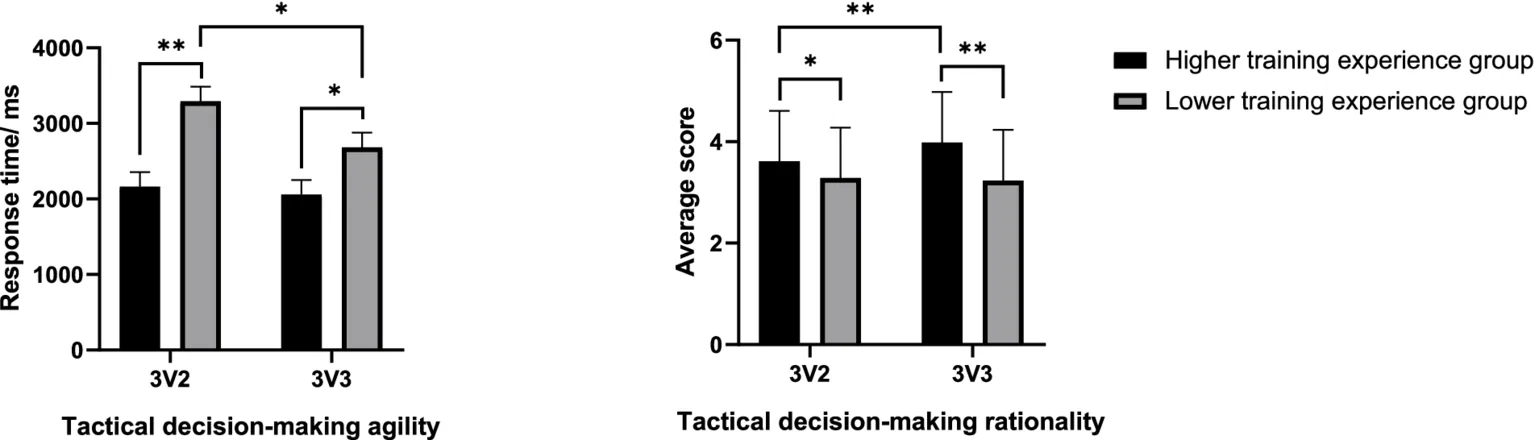
Results of a two-factor ANOVA on the efficiency of offensive tactical decision-making in soccer players. *p < 0.05; **p < 0.01; Error bars represent SD; the number of trials for the high training experience group and the low training experience group in 3V2 offensive tactical decision-making was 144 trials each; the number of trials for the high training experience group and the low training experience group in 3V3 offensive tactical decision-making was 144 trials each; subsequent figure notes follow the same convention.

### 3.2 Visual search data

#### 3.2.1 Goal area

In terms of the number of fixations in the goal area, the main effect of athletic level was not significant (*F* = 3.813, *p* = 0.053, *η_p_^2^* = 0.026); the main effect of stimulus material was also not significant (*F* = 0.065, *p* = 0.799, *η_p_^2^* < 0.001); and the interaction effect between athletic level and stimulus material was not significant (*F* = 0.837, *p* = 0.362, *η_p_^2^* = 0.006). Further simple effect analysis revealed that when watching the 3V3 video, the number of fixations of the higher training experience group (1.58 ± 0.80) was significantly higher than that of the lower training experience group (1.26 ± 0.51; *p* = 0.034; Cohen’s *d* = 0.477); when watching the 3V2 video, there was no significant difference in the number of fixations between the two groups (*p* > 0.05). Additionally, there were no significant differences in the number of fixations between watching 3V2 and 3V3 videos in both the higher training experience group and the lower training experience group (*p* > 0.05).

In terms of fixation duration in the goal area, the main effect of athletic level was significant (*F* = 15.740, *p* < 0.001, *η_p_^2^* = 0.099), with the higher training experience group exhibiting significantly longer fixation durations (704.57 ± 573.92 ms) compared to the lower training experience group (370.25 ± 212.99 ms). The main effect of stimulus material was not significant (*F* = 3.663, *p* = 0.058, *η_p_^2^* = 0.025); and the interaction effect between athletic level and stimulus material was also not significant (*F* = 2.603, *p* = 0.109, *η_p_^2^* = 0.018). Further simple effect analysis showed that when watching the 3V2 video, the fixation duration of the higher training experience group (536.77 ± 475.05 ms) was significantly shorter than that of the lower training experience group (359.27 ± 231.52 ms; *p* < 0.001, Cohen’s *d* = 0.475); when watching the 3V3 video, there was no significant difference in fixation duration between the two groups (*p* > 0.05). In addition, the fixation duration of the higher training experience group (536.77 ± 475.05 ms) when watching the 3V2 video was significantly shorter than that when watching the 3V3 video (802.72 ± 607.42 ms; *p* = 0.009, Cohen’s *d* = 0.488), while there was no significant difference in fixation duration of the lower training experience group between watching the 3V2 and 3V3 videos (*p* > 0.05). Detailed results are shown in Figure 3.

**Figure 3:**
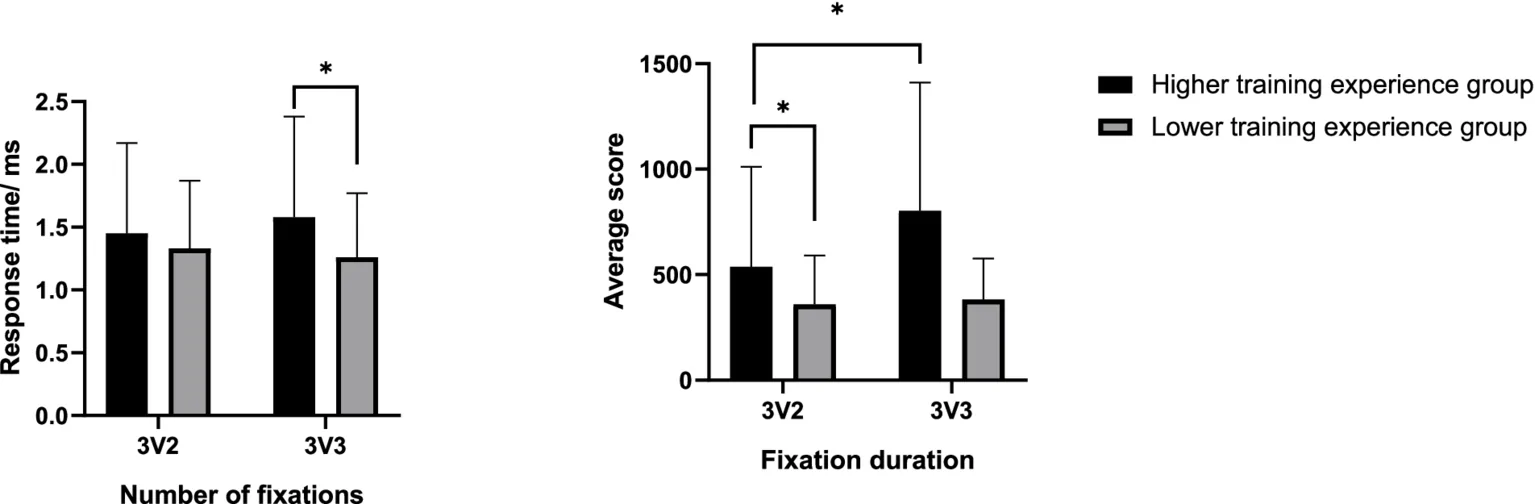
Results of a two-factor ANOVA on visual search characteristics of athletes in the goal area.

#### 3.2.2 Gap area

The main effect of athletic level on the number of fixations in the gap area is significant (*F* = 9.713, *p* = 0.002, *η_p_^2^* = 0.070). The number of fixations in the higher training experience group (1.54 ± 0.77) is significantly higher than that in the lower training experience group (1.16 ± 0.42). The main effect of stimulus materials is not significant (*F* = 2.422, *p* = 0.122, *η_p_^2^* = 0.018). The interaction between athletic level and stimulus materials is also not significant (*F* = 0.098, *p* = 0.754, *η_p_^2^* = 0.001). Further simple effect analysis showed that the number of fixations of the higher training experience group (1.44 ± 0.71) when watching the 3V2 video was significantly higher than that of the lower training experience group (1.04 ± 0.20; *p* < 0.05, Cohen’s *d* = 0.767); the number of fixations of the higher training experience group (1.58 ± 0.80) when watching the 3V3 video was also significantly higher than that of the lower training experience group (1.26 ± 0.51; *p* < 0.05, Cohen’s *d* = 0.477). However, there is no significant difference in the number of fixations between the higher training experience group and the lower training experience group when watching 3V2 and 3V3 videos (*p* < 0.05).

In terms of fixation duration in the gap area, the main effect of athletic level is significant (*F* = 19.853, *p* < 0.001, *η_p_^2^* = 0.132). The fixation duration of the higher training experience group (720.82 ± 575.97 ms) is significantly longer than that of the lower training experience group (320.57 ± 189.12 ms). The main effect of stimulus materials is also significant (*F* = 5.857, *p* = 0.017, *η_p_^2^* = 0.043). The fixation duration of participants while watching 3V2 videos (395.84 ± 377.05 ms) is notably shorter compared to that of 3V3 videos (647.43 ± 535.30 ms). The interaction between athletic level and stimulus materials is not significant (*F* = 0.529, *p* = 0.468, *η_p_^2^* = 0.004). Further simple effect analysis showed that the fixation duration of the higher training experience group (547.20 ± 467.69 ms) when watching the 3V2 video was significantly longer than that of the lower training experience group (244.48 ± 154.27 ms; *p* < 0.05, Cohen’s *d* = 0.869); the fixation duration of the higher training experience group (802.72 ± 607.42 ms) when watching the 3V3 video was significantly longer than that of the lower training experience group (381.94 ± 194.46 ms; *p* < 0.01, Cohen’s *d* = 0.933). The fixation duration of the higher training experience group when watching the 3V2 video (547.20 ± 467.69 ms) was significantly shorter than that when watching the 3V3 video (802.72 ± 607.42 ms; *p* = 0.009, Cohen’s *d* = 0.471), while there was no significant difference in the fixation duration of the lower training experience group between watching the 3V2 and 3V3 videos (*p* > 0.05). Detailed results are shown in Figure 4.

**Figure 4:**
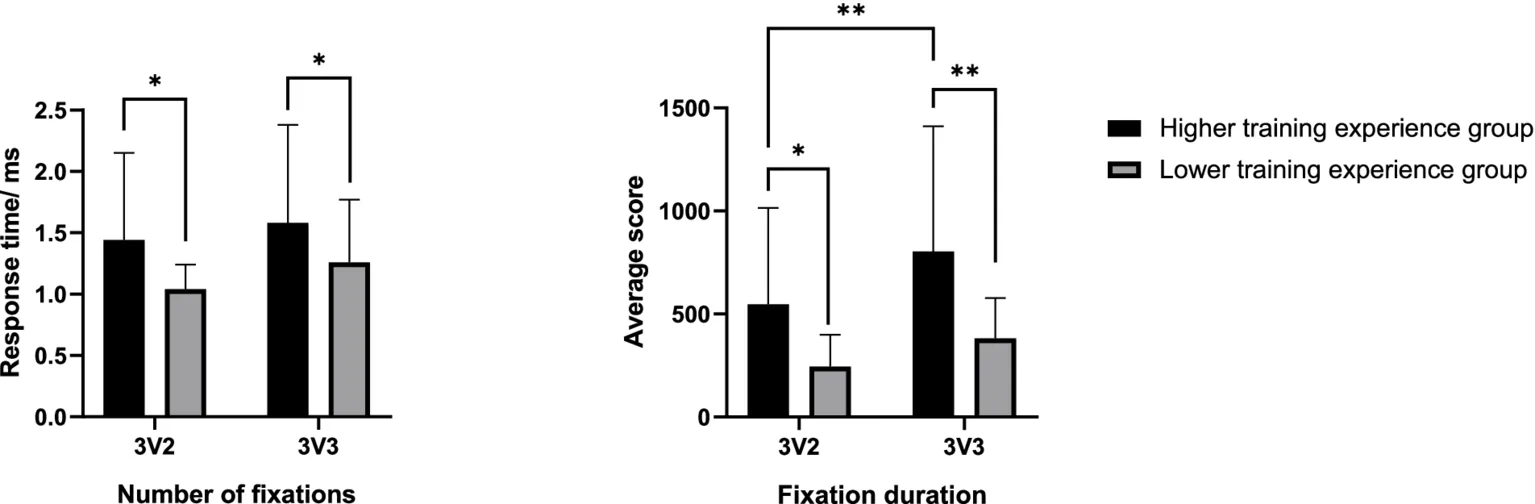
Results of a two-factor ANOVA on visual search characteristics of athletes in the gap area.

#### 3.2.3 Defensive player area

The main effect of the athletic level was not significant in terms of the number of fixations in the defensive player area (*F* = 0.614, *p* = 0.434, *η_p_^2^* = 0.003). The main effect of stimulus material was significant (*F* = 31.325, *p* < 0.001, *η_p_^2^* = 0.147), with participants showing significantly more fixations when watching the 3V2 video (2.94 ± 1.41) than the 3V3 video (1.96 ± 0.94). There was no significant interaction effect between the athletic level and stimulus material (*F* = 0.007, *p* = 0.934, *η_p_^2^* < 0.001). Further simple effect analysis showed that the number of fixations of the higher training experience group when watching the 3V2 video (2.88 ± 1.05) was significantly higher than that when watching the 3V3 video (1.89 ± 0.85; *p* < 0.05, Cohen’s *d* = 1.036); the number of fixations of the lower training experience group when watching the 3V2 video (3.00 ± 1.70) was significantly higher than that when watching the 3V3 video (2.04 ± 1.03; *p* < 0.05, Cohen’s *d* = 0.683). In terms of the fixation duration in the defensive player area, the main effect of the athletic level was not significant (*F* = 0.305, *p* = 0.581, *η_p_^2^* = 0.002). The main effect of stimulus material was also not significant (*F* = 0.130, *p* = 0.719, *η_p_^2^* = 0.001). There was no significant interaction effect between the athletic level and stimulus material (*F* = 0.197, *p* = 0.658, *η_p_^2^* = 0.001). Detailed results are shown in Figure 5.

**Figure 5:**
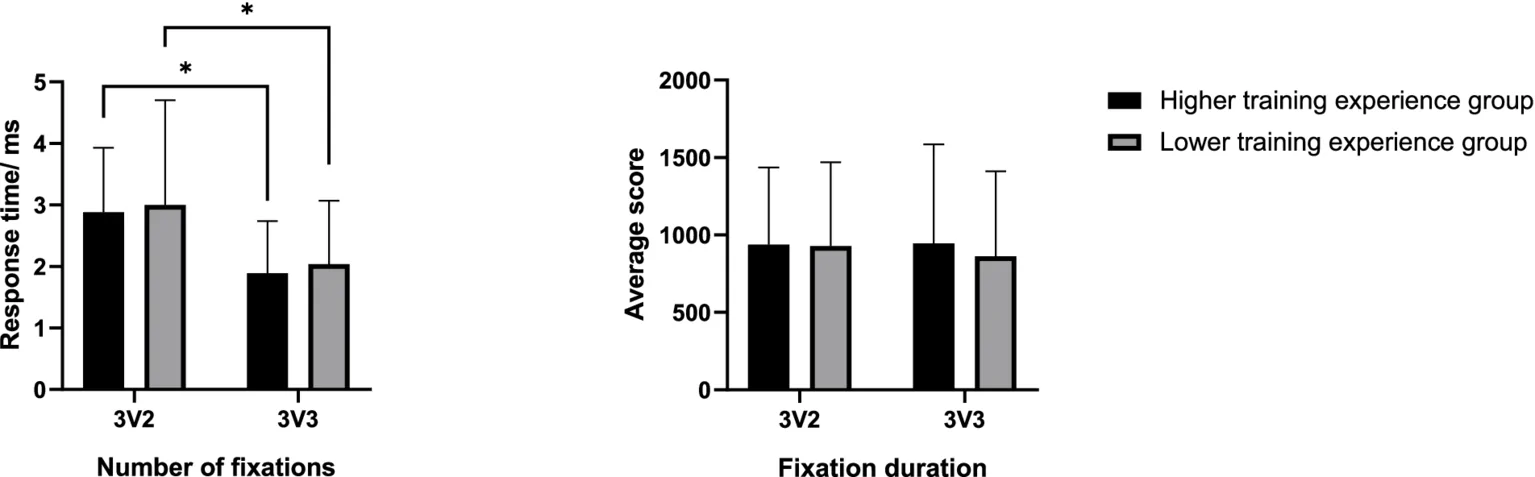
Results of a two-factor ANOVA on visual search characteristics of athletes in the defensive player area.

#### 3.2.4 Near offensive player area

The main effect of the athletic level was not significant in terms of the number of fixations on the proximal attacking player area (*F* = 0.178, *p* = 0.673, *η_p_^2^* = 0.001). However, the main effect of stimulus materials was significant (*F* = 4.355, *p* = 0.038, *η_p_^2^* = 0.023), with participants watching 3V2 videos (1.58 ± 0.99) fixating significantly fewer times than those watching 3V3 videos (1.89 ± 1.04). The interaction effect between athletic level and stimulus materials was not significant (*F* = 0.151, *p* = 0.698, *η_p_^2^* = 0.001). For the duration of fixations on the proximal attacking player area, the main effect of athletic level was significant (*F* = 4.171, *p* = 0.043, *η_p_^2^* = 0.022), with the higher training experience group (785.87 ± 725.92 ms) fixating significantly less time than the lower training experience group (981.33 ± 762.05 ms). The main effect of stimulus materials was also significant (*F* = 68.979, *p* < 0.001, *η_p_^2^* = 0.271), with participants watching 3V2 videos (518.24 ± 341.93 ms) fixating significantly shorter durations than those watching 3V3 videos (1291.73 ± 861.64 ms). The interaction effect between athletic level and stimulus materials was not significant (*F* = 0.287, *p* = 0.593, *η_p_^2^* = 0.002). Further simple effect analysis showed that the fixation duration of the higher training experience group when watching the 3V2 video (448.32 ± 298.28 ms) was significantly shorter than that when watching the 3V3 video (1169.45 ± 869.07 ms; *p* < 0.001, Cohen’s *d* = 1.110); the fixation duration of the lower training experience group when watching the 3V2 video (588.16 ± 370.51 ms) was significantly shorter than that when watching the 3V3 video (1408.70 ± 847.37 ms; *p* < 0.001, Cohen’s *d* = 1.255). Detailed results are shown in Figure 6.

**Figure 6:**
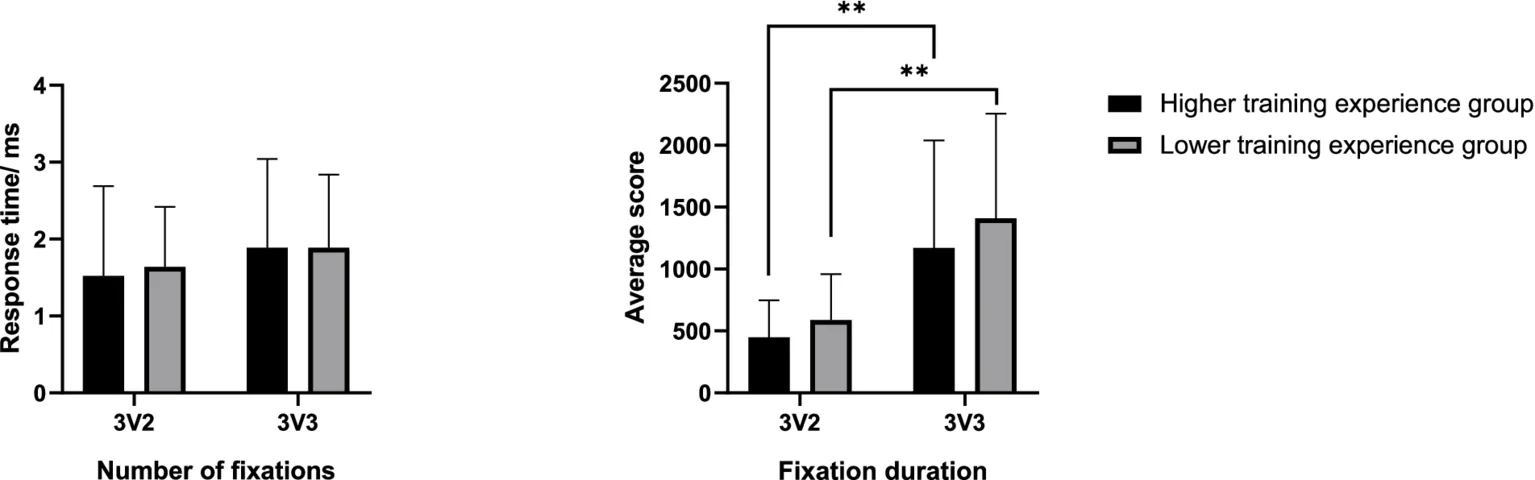
Results of a two-factor ANOVA on visual search characteristics of athletes in the near offensive player area.

#### 3.2.5 Far offensive player area

The main effect of athletic level was significant in terms of the number of fixations on the region of the distal attacking players (*F* = 8.269, *p* = 0.005, *η_p_^2^* = 0.053). The higher training experience group exhibited a significantly higher number of fixations (1.75 ± 1.03) compared to the lower training experience group (1.42 ± 0.72). However, the main effect of stimulus material was not significant (*F* = 0.379, *p* = 0.539, *η_p_^2^* = 0.003). A significant interaction effect was observed between athletic level and stimulus material (*F* = 6.011, *p* = 0.015, *η_p_^2^* = 0.039). Calculations yielded a post-hoc observed power of 0.286 for this interaction effect, indicating insufficient statistical power of the sample. Further simple effect analysis showed that when watching the 3V3 video, the number of fixations of the higher training experience group (2.04 ± 1.29) was significantly higher than that of the lower training experience group (1.25 ± 0.44; *p* = 0.001, Cohen’s *d* = 0.820); moreover, the number of fixations of the higher training experience group (2.04 ± 1.29) when watching the 3V3 video was significantly higher than that when watching the 3V2 video (1.58 ± 0.82; *p* = 0.032, Cohen’s *d* = 0.426).

In terms of fixation duration on the region of the distal attacking players, the main effect of athletic level was also significant (*F* = 9.326, *p* = 0.003, *η_p_^2^* = 0.059). The higher training experience group spent significantly longer fixating (783.79 ± 701.04 ms) than the lower training experience group (588.42 ± 455.38 ms). The main effect of stimulus material was also significant (*F* = 28.406, *p* < 0.001, *η_p_^2^* = 0.067), with the higher training experience group spending significantly less time fixating (511.33 ± 364.30 ms) compared to the lower training experience group (985.71 ± 788.06 ms). There was also a significant interaction effect between athletic level and stimulus material (*F* = 10.561, *p* = 0.001, *η_p_^2^* = 0.067). Calculations yielded a post-hoc observed power of 0.474 for this interaction effect, indicating insufficient statistical power of the sample. Further simple effect analysis showed that when watching the 3V3 video, the fixation duration of the higher training experience group (1266.86 ± 863.95 ms) was significantly longer than that of the lower training experience group (704.57 ± 568.19 ms; *p* < 0.001, Cohen’s *d* = 0.769); moreover, the fixation duration of the higher training experience group (1266.86 ± 863.95 ms) when watching the 3V3 video was significantly longer than that when watching the 3V2 video (502.00 ± 367.90 ms; *p* < 0.001, Cohen’s *d* = 1.152). Detailed results are shown in Figure 7.

**Figure 7:**
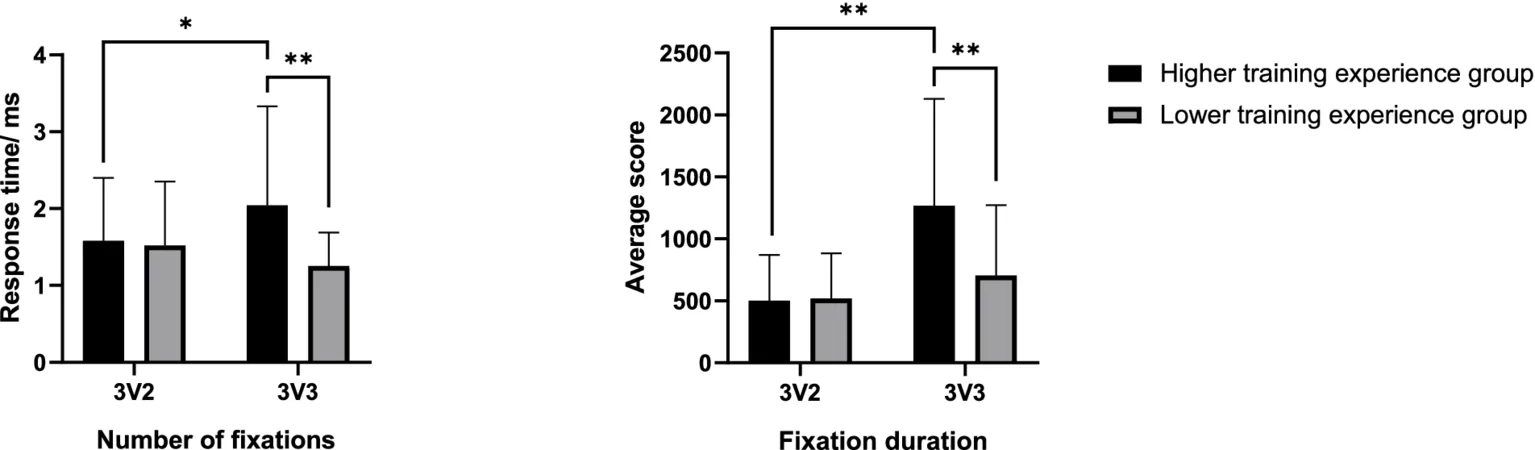
Results of a two-factor ANOVA on visual search characteristics of athletes in the far offensive player area.

## 4 Discussion

### 4.1 Higher training experience group demonstrated more efficient tactical decision-making performance

The results of this study revealed significant differences in the agility and rationality of tactical decision-making between the higher training experience group and the lower training experience group, wherein the higher training experience group demonstrated superior overall tactical decision making performance. All findings of this study are empirical results obtained under the first-person video simulation paradigm, which can reflect the differentiation of decision-making behaviors among players with different training experiences in local offensive scenarios. These findings are consistent with previous studies on soccer-specific decision-making behavior (Shi et al., 2023; Vítor de Assis et al., 2021). Within the present experimental paradigm, soccer players with extensive training experience, drawing on long-term accumulated tactical experience and knowledge reserves, are able to rapidly filter valid information from stimulus videos and achieve faster and more reasonable tactical decision-making (Shi et al., 2023).

In addition, this study also found that compared to the 3V2 offensive tactical decision-making task, the higher training experience group exhibited more reasonable tactical decisions in the 3V3 task. In the 3V3 scenario, due to the increase in the number of players on the field, tactical changes and detailed handling become even more crucial. Players need to simultaneously consider the positions, actions, and intentions of more teammates and opponents. Therefore, the higher training experience group may pay more attention to tactical details and variations, and make more cautious decisions in selecting their routes. Conversely, in the 3V2 scenario, with fewer players on the field and relatively larger gaps, players may be more inclined to take riskier routes, such as directly shooting or dribbling towards the gaps.

Meanwhile, the quantitative results of the present study revealed that the lower training experience group exhibited significantly longer decision-making response times under the 3V3 condition compared with the 3V2 condition. From the perspective of the informational characteristics of the experimental scenarios, 3V3 videos impose a higher tactical information load. Given the imperfect information-processing system of the lower training experience group, participants need to take abundant non-critical information into account during decision-making, which in turn slows down their overall decision-making efficiency.

### 4.2 Higher training experience group have a more cost-effective visual search pattern

In the 3V2 offensive tactical decision-making scenario, higher training experience group devote more fixation counts and durations to the goal AOI and the gap AOI. However, in the 3V3 offensive tactical decision-making scenario, experts pay more attention to the gap AOI and the far offensive player AOI, also increasing their fixation counts and durations. It should be noted that the post-hoc observed power for the group × scenario interaction effects for fixation count and fixation duration in the far offensive player AOI were both below the acceptable threshold, indicating insufficient statistical power for testing these interaction effects. Accordingly, conclusions regarding these interaction effects should be treated as tentative.

These observations indicate that regardless of the tactical decision-making scenario, the gap AOI is always a key area of focus for experts, consistent with previous research findings by Machado et al. (2017) and Shi et al. (2023). By observing and effectively utilizing the gap, teams can create offensive opportunities, improve shooting quality, disrupt defensive formations, enhance team collaboration, and ultimately increase game efficiency. These factors play crucial roles in tactical execution and game outcomes (Praça et al., 2015). It is worth noting that the size of the gap is not fixed; it dynamically adjusts as players from both teams interact and maneuver. This change contains rich tactical information, making it more likely to attract the attention of experienced experts. A study by Roca et al. (2018) also found that creative players tend to devote more fixation time to observing positions that contain more tactical information.

Moreover, under the 3V2 and 3V3 offensive tactical decision-making scenarios, the AOI that the higher training experience group pays attention to seems to be different. In the 3V2 offensive tactical decision-making scenario, the higher training experience group invests more fixations and gaze time in the goal area. The possible reason is that the goal is the key area for the expert group’s visual anchoring. In the 3V2 offensive situation, the offensive team has a significant advantage in numbers, and the gaps between defensive players are relatively large, so shooting may become the main choice for the higher training experience group. However, in the 3V3 offensive tactical decision-making scenario, the higher training experience group invests more fixations and gaze time in the far offensive player area. The possible reason is that in the face of relatively dense defense with an equal number of players, the difficulty of direct shooting for the higher training experience group increases, so they tend to prioritize passing the ball to the far offensive players, thereby increasing the threat to the goal. The results of this study are also supported by previous studies (Natsuhara et al., 2020; Zhang et al., 2004, 2005). For example, Zhang et al. (2004, 2005) studied the decision-making behavior of basketball players in sports situations and found that the higher training experience group paid more attention to supporting players and gap areas.

Compared with the 3V2 scenario, the higher training experience group demonstrated a significant increase in fixation duration during the 3V3 offensive tactical decision-making task. This change was evident across the goal, gap, near offensive player, and far offensive player areas. From the perspective of Cognitive Load Theory (Shi et al., 2023), the implications of fixation duration should be interpreted differently across scenarios. Specifically, in the less-demanding 3V2 scenario, the relatively shorter fixation duration of the higher training experience group reflects their efficient information-encoding capacity. By contrast, in the high-complexity 3V3 scenario, the prolonged fixation duration represents players’ adaptive allocation of cognitive resources to meet elevated task demands, alongside deep processing of key tactical information. The 3V3 offensive scenario is substantially more difficult than 3V2, with greater volumes of tactical information available for processing and increased challenges in extracting valid information. Accordingly, players need to invest more fixation duration to analyze and evaluate on-field situations. Even under such challenges, the higher training experience group can still accurately identify critical areas such as gaps and make efficient and economical decisions accordingly.

Relying on the strictly-controlled first-person video simulation paradigm, the present study quantitatively verified the differentiated performance of the above-mentioned visual-search behaviors under varying levels of tactical complexity. Regardless of whether it’s a 3V2 or 3V3 scenario, the visual search advantages of soccer players are evident in the following aspects. Firstly, players need to effectively allocate their attention, focusing on the position and dynamics of gaps while also keeping track of other areas on the field. This ability enables players to maintain a clear mind in complex tactical environments and make accurate decisions. Secondly, soccer players need to quickly process the large amount of information obtained from visual search. They can identify key tactical elements, such as passing routes and running directions, within a short time and react promptly. Finally, through visual search, players are able to predict the actions of opponents and teammates in advance, which help them, respond earlier and anticipate more effectively in the game.

### 4.3 Theoretical contributions of this study

Based on the controlled experimental paradigm of 3V2 and 3V3 local offensive soccer scenarios, the present study provides contextualized empirical evidence for the application of multiple objects tracking theory within the field of sport cognition. Overcoming the limitation of difficult AOI delineation in 11-a-side match contexts, this study systematically analyzes multi-target information processing patterns in local tactical scenarios and reveals the differentiated quantitative performance of players’ multiple objects tracking behaviors under changing tactical complexity. These findings can serve as a reference for refining the theoretical system of multi-target processing in sport cognition. Meanwhile, this study helps to further enrich the explanatory dimensions of cognitive load theory regarding visual search differences among athletes. Cognitive load theory suggests that task difficulty is associated with cognitive resource consumption to a certain extent. Based on a comparative analysis of visual search behaviors between experts and novices in two tactical scenarios, this study attempts to construct a differential explanatory framework of “cognitive load-visual strategy matching”, providing new insights for relevant theoretical interpretations in future research.

### 4.4 Practical value of this study

Firstly, this study preliminarily identified the core visual attention areas of the high training experience group under different levels of tactical complexity, which can provide referable training targets for targeted cognitive training. Coaches may refer to the visual search characteristics of athletes with extensive training experience and attempt to integrate a gaze allocation guidance module into training. For instance, key areas of interest can be marked via video replay, requiring athletes to actively organize their observation priorities in simulated situations (e.g., open spaces, positions of far-side teammates). Meanwhile, specialized training tasks for gap utilization can be designed. For example, temporary defensive gaps can be artificially created in 3V3 training to guide athletes to quickly identify gaps and exploit them through reasonable decisions (passing, dribbling, etc.), which may help improve decision-making rationality and tactical execution efficiency.

Secondly, this study attempts to provide quantifiable cognitive evaluation indicators for athlete selection. In youth talent identification, the 3V2 and 3V3 experimental scenarios adopted in this study can be applied to examine candidates’ gaze preferences for key areas of interest. Combined with decision-making response time and rationality scores, this approach helps to screen athletes with high cognitive efficiency and decision-making potential, and to a certain extent compensates for the limitations of traditional selection models that overemphasize physical fitness and technical skills while neglecting cognitive abilities.

Finally, this study offers targeted practical pathways for improving athletes’ cognitive abilities. Based on the cognitive differences between athletes with high and low training experience, this research preliminarily indicates the optimization direction of athletes’ cognitive capacity, helping to break the inherent bottleneck of “valuing skills over cognition” in traditional training. On the one hand, dynamic video clips of 3V2/3V3 tactical scenarios can be presented, requiring athletes to mark key areas (open spaces, far attacking teammates, etc.) within a specified time. With real-time feedback on gaze deviations provided by eye-tracking devices, inefficient visual search habits can be gradually corrected, and an efficient information-filtering pattern similar to that of highly trained athletes can be gradually formed. On the other hand, visual-decision linkage training can be explored: in simulated scenarios, athletes first observe tactics and report their visual focus, then explain their decision-making logic based on such focus. Through the linkage training of “visual focus → decision-making logic”, the coordination between the two processes can be strengthened, which helps to improve the efficiency of the “rapid observation-rational decision” linkage in real competitions.

### 4.5 Limitations of this study

Firstly, regarding the authenticity of scenario reproduction, on the one hand, the experimental scenarios were recreated by student athletes, and there are objective differences in their technical proficiency and depth of tactical understanding compared with professional players, which may reduce the authenticity of the scenario reproduction; on the other hand, the static viewing environment in the laboratory lacks dynamic interference factors present in real matches (such as physical confrontation, physical exertion, match pressure, and crowd noise), making it difficult for participants to develop an immersive sense of decision-making engagement. In addition, while this study focuses on objective eye-movement indicators, it should be acknowledged that tactical decision-making is also influenced by players’ first-person subjective experience of match situations. Secondly, regarding the scope of sample coverage, the participants in this study were only male athletes, with no inclusion of female athletes. Since athletes of different genders may differ in visual search habits, tactical decision-making preferences, and other aspects, focus solely on male athletes limits the generalizability of the research conclusions. As a result, the conclusions cannot fully reflect the visual search characteristics of the broader soccer athlete population. In addition, the two groups were not perfectly matched for age, which may introduce confounding developmental variables. Finally, regarding the limitations of the research scenarios, this study only explored the visual search characteristics of soccer athletes in two small-scale offensive tactical decision-making scenarios: 3V2 and 3V3. However, a real soccer match is a full-pitch team sport involving multiple scenario transitions, covering complex situations such as offensive- defensive transitions, set pieces (e.g., corner kicks, free kicks), and long-range switching attacks. The singularity of the current research scenarios fails to fully cover the tactical dimensions of real matches. Therefore, further expansion of scenario types is needed to explore a more comprehensive set of visual search patterns.

In view of the above-mentioned limitations of this study, the following future prospects are proposed. Firstly, virtual reality technology can be used to replicate the tactile sensation of physical confrontation in real matches, and progressive muscle load can be applied through equipment to simulate physical exertion. This allows participants to complete decision-making tasks in an immersive experience, enhancing their sense of engagement in decision-making and making the research results more consistent with the cognitive state in real soccer matches. Meanwhile, future research may combine eye-tracking with stimulated-recall interviews to explore athletes’ subjective reasoning processes and situational awareness, so as to provide a more comprehensive account of sport-expertise performance. Secondly, future research should recruit female soccer athletes as participants. Efforts should be made to achieve group-level matching of demographic variables such as age during participant recruitment, so as to explore the mechanisms through which gender and age influence the cognitive processes underlying soccer tactical decision-making. This will address the current gaps in sample coverage and reduce interference from development-related confounding factors. Thirdly, during the AOI marking stage, a dual verification method combining expert judgment and algorithmic analysis can be adopted. First, soccer tactical experts label the AOIs in the experimental videos. Then, computer vision algorithms are used to compare the overlap rate and dynamic change errors between the experimentally labeled AOIs and those in the original matches. AOIs with excessive deviations are corrected to minimize consistency biases in key indicators of the stimulus materials, providing reliable data support for the accurate analysis of visual search characteristics. Finally, future research should establish a comprehensive tactical decision-making research system that goes beyond the limitations of small-scale scenarios such as 3V2 and 3V3. It should incorporate complex scenarios that frequently occur in real matches, such as full-pitch offensive-defensive transitions and counterattacks, to explore the patterns of how athletes adjust their visual search strategies in response to different game situations. This will help cover the full tactical dimensions of real soccer matches and reveal more comprehensive visual search laws of athletes.

## 5 Conclusion

The difference between the visual search characteristics of the higher and lower training experience group is caused by the amount of stimulus information received and the different cognitive strategies. Higher training experience group have efficient tactical decision making compared to the lower training experience group. In the 3V2 offensive tactical decision-making scenario, the higher training experience group invests more fixations and gaze duration in the gap and the goal AOI. While in the 3V3 offensive tactical decision-making scenario, the higher training experience group allocates more fixations and gaze duration in the gap and far offensive player AOI. As the difficulty of stimulus materials increases and the difficulty of cognition increases, higher training experience group increase the number of fixations in key areas and extend the fixation duration to obtain accurate information. However, it should be noted that the post-hoc observed power for the group × scenario interaction effects regarding fixation count and fixation duration in the far offensive player AOI is low, which warrants further validation with a larger sample size.

## Data Availability

The raw data supporting the conclusions of this article will be made available by the authors, without undue reservation.
